# Amplified spontaneous emission from all-inorganic perovskite on a flexible substrate with silk fibroin

**DOI:** 10.1038/s41598-022-12313-2

**Published:** 2022-06-16

**Authors:** Chin-Yi Yang, Liang-Yu Jian, Yi-Ting Lee, Zong-Liang Tseng, Ja-Hon Lin

**Affiliations:** 1 Department of Dermatology, Chang Gung Memorial Hospital, Linkou Branch, Taoyuan, Taiwan; 2 Department of Electro-optical Engineering, National Taipei University of Technology, Taipei, 10608 Taiwan; 3 Center for Organic Photonics and Electronics Research (OPERA), Kyushu University, Fukuoka, 819-0395 Japan; 4 Department of Electronic Engineering and Organic Electronics Research Center, Ming Chi University of Technology, New Taipei City, 243303 Taiwan; 5 Department of Dermatology, New Taipei Municipal TuCheng Hospital, New Taipei City, Taiwan

**Keywords:** Lasers, LEDs and light sources, Optical physics

## Abstract

Stretchable microcavity lasers reveal potential application in flexible displays, biomedicine, and wearable devices in the near future. In this work, we investigated the characteristic of amplified spontaneous emission (ASE) from all inorganic CsPbBr_3_ QDs on a flexible PET substrate with the assistance of biocompatible silk fibroin (SF) film. In comparison with the sample on PET directly, the ASE of all-inorganic perovskite film revealed a lower threshold of 32.7 μJ/cm^2^, higher slope efficiency, and a larger gain coefficient of around 100.0 cm^−1^ owing to the better stack and good arrangement of the CsPbBr_3_ QDs on top of the SF film. For the temperature-dependent ASE measurement, the larger characteristic temperature of around 277 K is obtained from CsPbBr_3_ QD/SF film, and the emission peak reveals a slight shift with temperature variation, which indicates its temperature-insensitive property. As the curvature of flexible substrate increases under the mechanical bending, the lasing threshold of CsPbBr_3_ QD/SF film was reduced along with the increase in slope efficiency owing to the enhancement in the index guiding effect.

## Introduction

Recently, low cost solution processing all-inorganic perovskites (CsPbX_3_, X = I, Br, and Cl), have become a revolutionary material to produce versatile optoelectronic devices such as photovoltaics^1^, light-emitting diodes (LEDs)^2^, sensors and photodetectors^3^ with outstanding performance. In contrast to perovskite bulks or films, all-inorganic perovskite quantum dots (QDs)^4^ have attracted significant attention owing to their superior properties such as high photoluminescence (PL), quantum yields (QYs)^5^, variable bandgap to shift the emission spectrum^6^, long diffusion length^7^, and narrow emission linewidth^8^. Besides, all-inorganic CsPbBr_3_ QDs have become the promising optical gain materials for the generation of amplified spontaneous emissions (ASE), because of their high PLQY and binding energy, and the production of microcavity lasers^9,10^ with assistance of optical cavities^10^. In 2015, Yakunin et al.^4^ demonstrated that the emission spectrum of room temperature (RT) ASE from CsPbX_3_ (X = Cl, Br or I, or mixed Cl/Br and Br/I systems) QD thin films cover almost entire visible spectrum range (440–700 nm).

Although all-inorganic perovskites reveal various impressive performances, many efforts demanded to improve their chemical and optical stabilities, and to reduce their moisture sensitivities of CsPbBr_3_ QDs and nanocrystals through different kinds of passivation techniques. For example, the ligand-modification strategy^11,12^ has been used to produce relatively long-term stable ASE at RT. Other methods include embedding CsPbBr_3_ QDs into the glass substrate^13^ or water less silica spheres^14^ to enhance their moisture resistance, which results in striking suppression of the blinking and elongation of the photon lifetime. A simple hydrophobic functionalization of the substrates with hexamethyldisilazane (HMDS) has been proposed to increase ASE operation stability of CsPbBr_3_ nanocrystal films up to 14 times^15^. Scientists are also interested in the production of random lasers (RLs) from all-inorganic perovskite based on the recurrent light scattering within aggregated QD films^16^. In order to depress optical degradation and increase the performance of RLs, the perovskite QDs were amino-mediated anchored onto the surfaces of monodisperse silica^17^ or dispersed among robust TiO_2_-based glass^18^.

Recently, biocompatibility and biodegradability devices from natural materials have revealed high potential in various applications. Owing to its remarkable optical characteristics, mechanical properties and chemical stability, biodegradable silk fibroin (SF) has been fabricated into diverse optical components such as waveguides, microlens arrays and photonic crystals, for specific application. The SF can be easily extracted from Bombyx mori cocoons and further manufactured into different forms such as hydrogels^19^, microspheres^20^, porous 3D matrices^21^, fibers^22^, and films^23^. On the other hand, one and three dimensional periodic structures, such as the SF grating replica^24^ and the inverse opal^25^ have been fabricated such that they can also combine with the laser dye as a gain medium to produce a distributed feedback (DFB) laser from SF nanograting^26^ and a RL from the 3D porous structure^27^.

Generally, the stretchable microcavity laser on a bendable substrate may find use in flexible displays, biomedicine diagnosis^28^, and wearable devices. Through mechanical stretching on a flexible substrate, the emission spectrum, the mode number^29^, and the polarization^30^ of the laser can be manipulated. A feasible and reliable approach for generating wide range wavelength tunable plasmonic random lasers^30,31^ has been reported by incorporating R6G laser dye as a gain medium with silver nanowires^30^ or silver nanoprisms^31^ onto a flexible silicone rubber slab and polyethylene terephthalate (PET) substrate, respectively. Through soft lithography by casting polydimethylsiloxane (PDMS) from a lotus leaf, Li et al.^32^ realized lotus-leaf-inspired flexible RL with a 14 nm tuning range under the bending condition. On the other hand, the stretchable microcavity laser has also been verified from organic-inorganic perovskite using the bendable cholesteric liquid crystal reflectors^33^ or the DFB structure. The lower threshold RL from CH_3_NH_3_PbBr_3_ thin film^34^ was achieved by the increase in the local curvature of a polyimide (PI) substrate to enhance the light scattering strengths. However, the ASE characteristic of all organic CsPbBr_3_ QDs on a flexible substrate has seldom been investigated. In this work, we studied the lasing performance of CsPbBr_3_ QD films on a bendable PET substrate under mechanical bending. In order to improve the surface quality and arrangement of CsPbBr_3_ QDs on a flexible substrate to enhance the efficiency and thermal stability of ASE, the bio-degradable SF film was inserted between the perovskite film and the PET.

## Sample preparation and experimental setup

**Figure 1:**
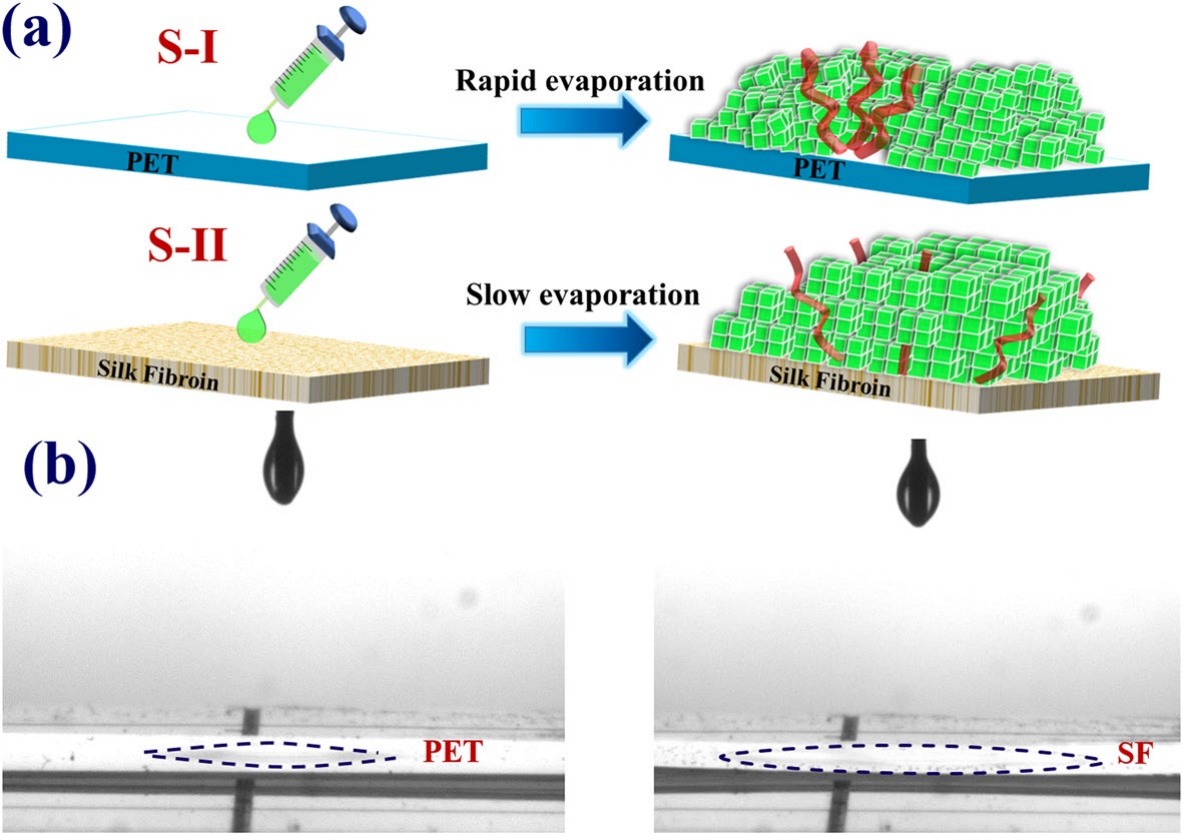
(**a**) Schematic diagram to illustrate spin-coating of CsPbBr_3_ QDs on substrates without (top) and with SF (bottom), which show the irregular and regular alignment of QDs, respectively, (**b**) Photographs of PET substrates without (left) and with (right) SF after a drop of 50 μL of hexane.

The CsPbBr_3_ QDs were prepared by the hot injection method^4^. First, the precursor I (pre-I) was synthesized by dissolving caesium carbonate (Cs$_{2}$CO_3_, 0.2442g) in 1-octadecene (ODE, 12mL) and oleic acid (OA, 0.75 mL) and then heated to 150 °C to ensure the total reaction. On the other hand, the precursor II (pre-II) was prepared by dissolving PbBr$_{2}$ (0.188 mmol) into dry 1-octadecene (ODE, 10 mL). After adding OA (1 mL) and oleylamine (OAM, 1 mL) at room temperature, the pre-II was heated to 120 °C. About 30 mins latter, the temperature of pre-II was further increased to 155 °C. The pre-I (0.8 mL) was cooled down to 100 °C and quickly injected into the pre-II to initialize the CsPbBr_3_ QDs. When the color turned green, the mixtures were immersed in an ice bath to terminate the reaction. Following this, the CsPbBr_3_ QDs were treated by a typical ligand reduction and were dispersed in hexane. The first purification centrifugation was carried out by the polar solvent ethyl acetate with ratio 1:1 (original solvent: polar solvent). The precipitated QDs in the bottom of tube were taken out for the second purification centrifugation by the N-hexane and ethyl acetate with a ratio of 2:3. In this work, two different kinds of CsPbBr_3_ QD films were prepared on the flexible polyethylene terephthalate (PET) substrate without (S-I) and with (S-II) SF film, respectively. For sample S-I (top in Fig. 1a), the dispersed CsPbBr_3_ QDs in hexane were spin coated directly on top of the PET substrate. For sample S-II (bottom in Fig.1a), the silk fibroin (SF), extracted from silkworm cocoons^19^, was deposited on top of a flexible PET substrate first by the drop-coating technique. The preparation of the aqueous SF solution involved three steps: degumming, dissolution in lithium bromide (LiBr) aqueous solution, and dialysis. First, Bombyx mori cocoon were boiled in a sodium carbonate (Na_2_CO_3_) solution to remove the sericin protein. The residual fibroin fibers were subsequently rinsed in distilled (DI) water and dried in the oven. To obtain an aqueous fibroin solution, the dried fibroin fibers were dissolved in a LiBr solution at 60 °C for 4 hours. Finally, we obtained the pure fibroin solution by the dialysis, using the dialysis membrane, at room temperature for two days. After drying at room temperature, the CsPbBr_3_ QDs were spin-coated on top of the SF film. Due to the low boiling point of hexane (69 °C), the hexane molecules evaporate quickly after spin-coating of CsPbBr_3_ QDs on a PET substrate for sample S-I which leads to the irregular stack of QDs (top in Fig.1a). On the contrary, for sample S-II, the SF film with a porous surface can efficiently capture the hexane molecules, resulting in a slow evaporation rate^35^. Thus, the QD films have more time to aggregate and align regularly on top of SF film (bottom of Fig. 1a). Figure 1b show the spreading of the hexane drop on the PET (Left) and SF film (right). The dispreading area (dashed line) of the blotting on SF film is larger than that in PET substrate, which is attributed to capillary phenomenon from the porous surface of the sample S-II.

**Figure 2:**
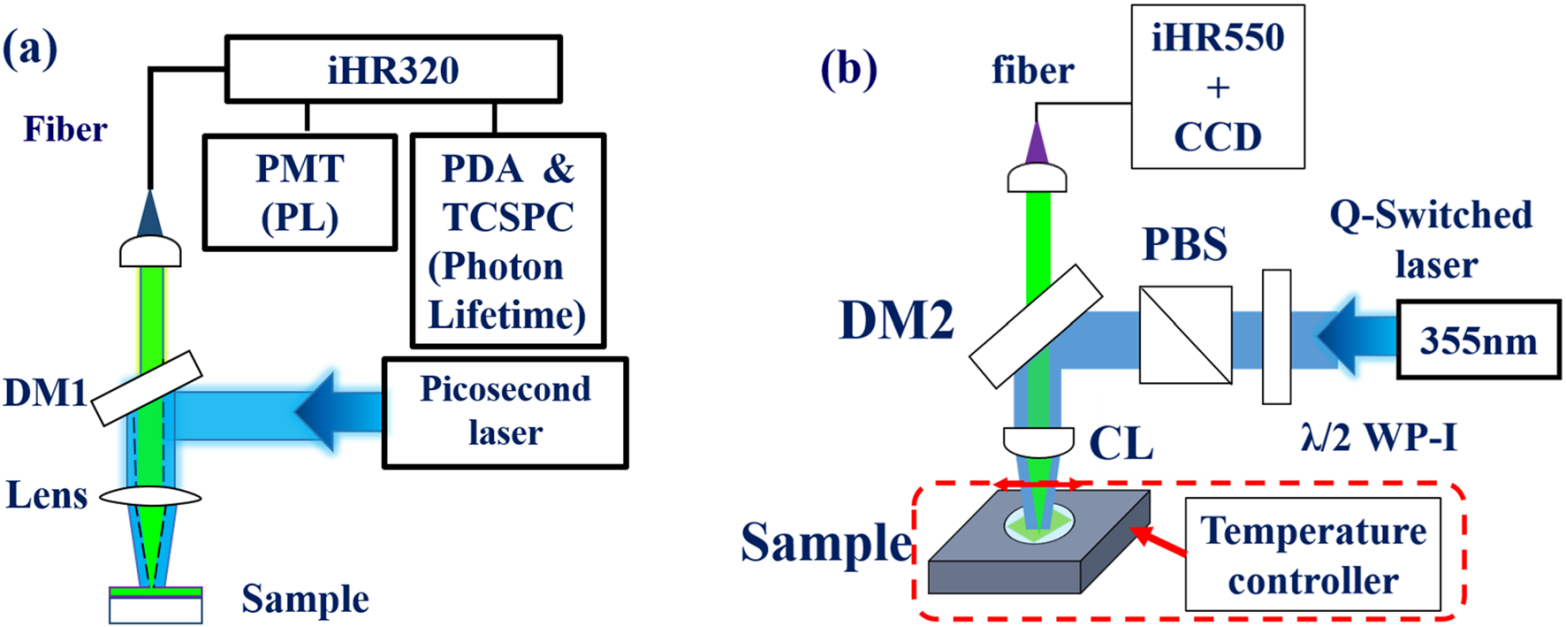
Schematic setup of (**a**) photoluminescence (PL), and (**b**) temperature-dependent ASE and measurement of CsPbBr_3_ QDs on flexible PET substrate.

In Fig. 2a, the micro-PL and photon decay trace of the sample were measured using a 372 nm picosecond pulsed laser as an excited source (PicoQuant Inc.) with 10 MHz repetition rate, and 50 ps pulse duration. The incident pump pulse was reflected by a dichroic mirror1 (DM1, LP02-407RU-25, Semrock Inc.) and then focused by an objective lens (10 ×). The emission light from the sample was collected by the objective lens and then passed through DM1. Finally, the emission spectrum was measured by a spectrometer (iHR320, Horiba Inc.) equipped with a photomultiplier tube (PMT, Hamamatsu Inc.). On the other hand, the photon lifetime was measured by time-correlated single photon counting (TCSPC, TimeHarp 260, PicoQuant Inc.) in combination with a photomultiplier detector assembly (PDA, PicoQuant Inc.) for recording the decay trace. The absorption spectrum was obtained using the deuterium lamp as a light source (DH-2000-BAL, Ocean Optics Inc.) and measured by a spectrometer (Flame-SUSVIS, Ocean optics Inc.). In Fig.2b, the ASE of CsPbBr_3_ QD films was generated by using a frequency-tripling Q-switched Nd:YAG laser ($\lambda$ = 355 nm, 7 ns duration, 10 Hz repetition rate) as an excited light source. The incident laser beam was reflected by a dichroic mirror2 (DM2, LP02-355RU-25, Semrock Inc.). A $\lambda$/2 plate and polarization beam splitter (PBS) were used to control the pulse energy. The pump pulse was focused onto the sample by a spherical lens (SL) with a focus length of 3.5 cm (effective area $\sim$ 0.0206 mm$^2$). The surface normal emission of the CsPbBr_3_ QDs film was collected by an optical fiber and measured by a monochromator (iHR 550, Horiba Inc.) equipped with an electrically cooled charge coupled device (CCD) (Syncerity, Horiba Inc.). In order to obtain the ASE of the sample under the bending condition, mechanical stress was applied to the film. Temperature-dependent ASE measurement of perovskite film can provide worthy information to investigate the exciton-phonon interaction and understand the temperature-related emission properties of lasers^36,37^. Here, the precise temperature of the CsPbBr$_3$ QD film from 78 K to 300 K was thermal controlled by the cryogenic system (ST-500-UC, Janis Inc.).

## Results and discussion

**Figure 3:**
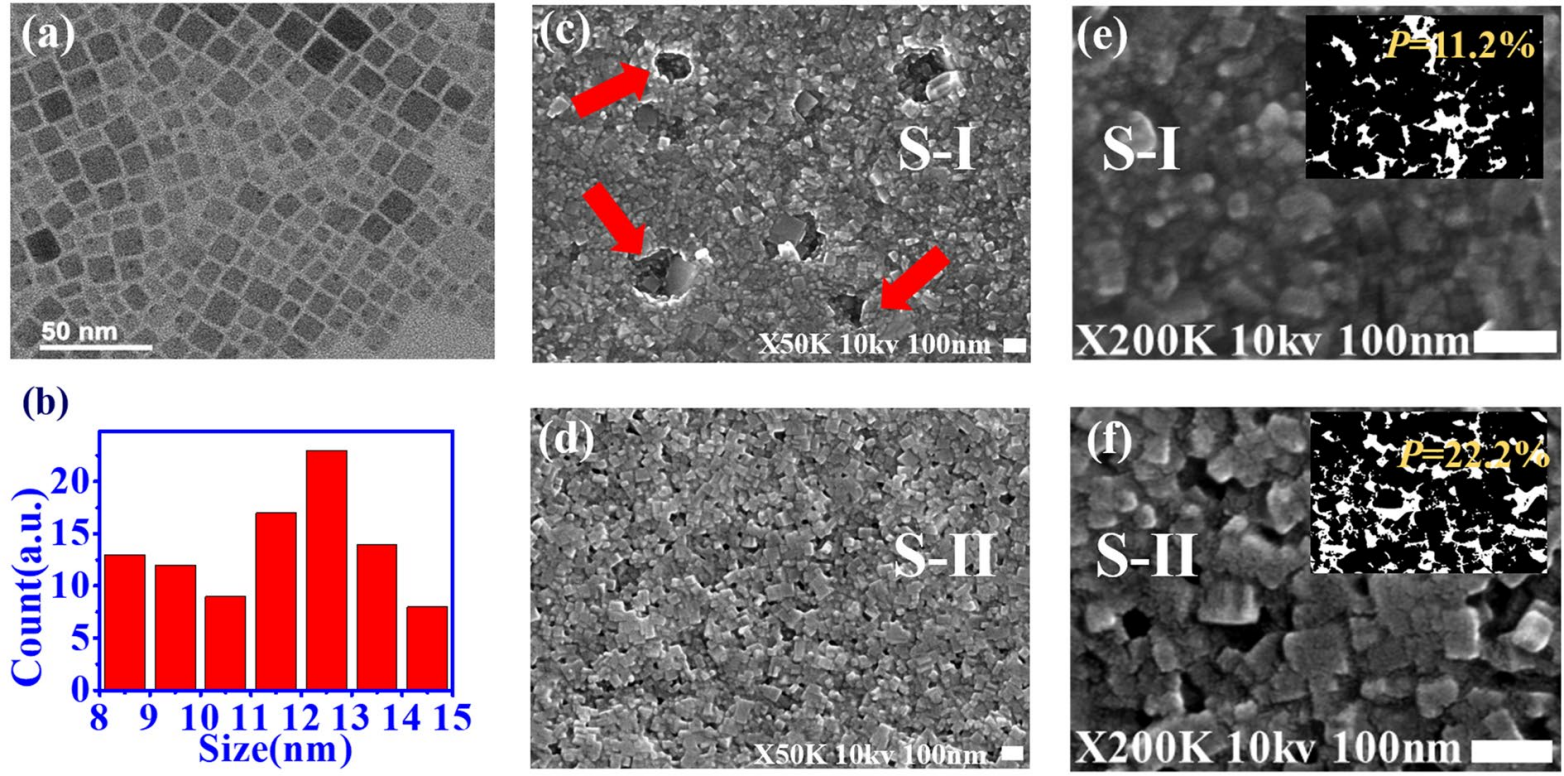
(**a**) TEM image, and (**b**) size distribution of CsPbBr_3_ QDs. SEM images of CsPbBr_3_ QD film on the PET substrate with 50K amplification (**c**) without and (**d**) with SF, and with 200k amplification (**e**) without and (**f**) with SF (Inset: estimated porosity image).

Figure 3a and b show the TEM image and the size distribution of the synthesized CsPbBr_3_ QD films, which illustrates the size variation of the QDs from 8 to 15 nm. The surface morphology of the perovskite film in the scanning electron microscope (SEM) images reveals the quite different arrangement of the QDs for samples S-I (without SF) and S-II (with SF). In Fig. 3c, the synthesized CsPbBr_3_ QD film without SF (S-I) reveals rough surface with a number of holes (red arrow). On the contrary, in Fig. 3d, sample S-II reveals a smooth surface and the uniform size of the aggregated CsPbBr_3_ QDs on the SF film. The amplified SEM image (× 200 k, Fig. 3f) also illustrates that the stack of QDs on the SF reveals regular alignment and better orientation than that on PET (Fig. 3e).

**Figure 4:**
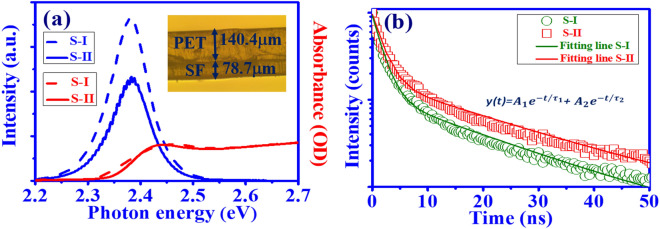
(**a**) PL spectra of S-I (W/O SF: dashed blue line) and S-II (W/ SF: solid blue line). The inset figure shows the optical microscopy image of S-II, (**b**) Photon decay trace of S-I (open green circles) and S-II (open red squares) and the corresponding fitting curves (solid line).

Figure 4a shows the absorption (red line) and PL spectra (blue line) of sample S-I (W/O SF) and S-II (W/SF). The figure indicates that the free exciton emission peaks of both samples are around 520 nm (2.38 eV), and the absorption peak is around 514 nm (2.41 eV). Owing to the resolution limit of measurement, the absorbance of two samples is close. However, the sample S-I (blue dash line) reveals stronger emission peak intensity than sample S-II (blue solid line) from micro-PL measurement. It is attributed to the high density of perovskite because of more small size aggregated CsPbBr_3_ QDs on the PET substrate. In order to demonstrate this point, the porosity of the two samples was calculated to be 11.2% and 22.2%, respectively, by using image analysis (Inaet of Fig. 3c,d). Here, the porosity is defined as P =$\sigma$ $_a$/$\sigma$ $_t$ from the SEM image, where $\sigma$ $_a$ is the area of porous regions, and $\sigma$ $_t$ is total area for the QD films. The lower porosity for sample S-I means higher QD density, which will lead to more excited excitons emitting light at the same pump irradiation. Besides, the spontaneous emission of sample S-II shows obvious interference fringes with the spacing $\Delta \lambda \sim$ 1.33 nm because of the Fabry–Perot (F–P) effect from the SF film. By the formula $\Delta \lambda = \lambda ^2$/2*nL*, *n* = 1.54^38^, the thickness *L* of silk film was estimated to be around 80.3 $\mu$m, which is close to the value measured from optical microscopy (Inset of Fig. 4a).

Figure 4b shows the logarithm scale photon decay traces of CsPbBr$_3$ QDs at 520 nm by the TCSPC measurement. Through the biexponential fitting, we obtain the fast decay time constant ($\tau _1$), around 1.70 ± 0.01 ns (samples S-I) and 1.84 ± 0.01 ns (sample S-II), resulting from the Auger recombination, and slow decay time constant ($\tau _2$), around 18.60 ± 0.03 ns (samples S-I) and 20.88 ± 0.02 ns (sample S-II), resulting from the single-exciton recombination of the CsPbBr$_3$^16,39^. In addition, the average PL lifetime $\tau _{ave}$ was calculated by the formula^9^:

1 $$\begin{aligned} \tau _{avg}=\Sigma _{i=1}^{n}\frac{A_{i}\tau _{i}^{2}}{A_{i}\tau _{i}}=\frac{A_{1}\tau _{1}^{2}+A_{2}\tau _{2}^{2}}{A_{1}\tau _{1}+A_{2}\tau _{2}} (n=2). \end{aligned}$$

According to Eq. (1) and considering the weight ratios of two samples, i.e, $A_1$= 87.11$\%$, $A_2$ = 11.88$\%$ for sample S-I and $A_1$= 83.23$\%$, $A_2$ = 16.77$\%$ for sample S-II, $\tau _{avg}$ of all-inorganic perovskite film with SF of about 15.13 ± 0.01 ns is larger than that without SF which is about 9.03 ± 0.01 ns.

**Figure 5:**
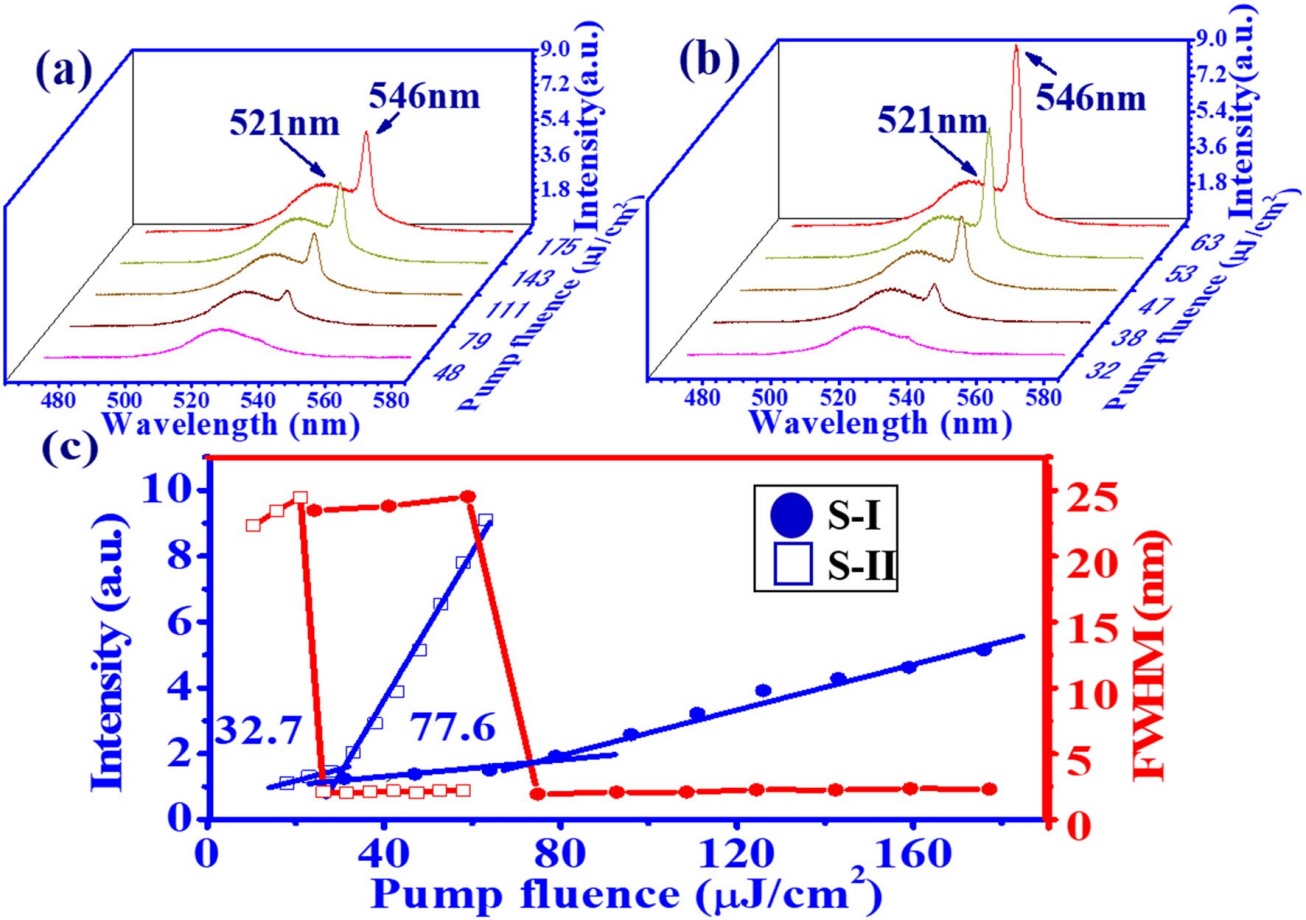
ASE spectra of CsPbBr_3_ QDs on the PET substrate for samples (**a**) S-I (W/O SF) and (**b**) S-II (W/ SF). (**c**) Peak intensity (red symbol line) and FWHM (blue symbol line) of ASE from the CsPbBr_3_ QD film for samples S-I (W/O SF) and S-II (W/ SF).

Figure 5a and b show the evolution of the ASE spectra from samples without and with SF as a function of pump fluence. At lower pump fluence, the emission spectrum is dominated by the spontaneous emission and shows the broad bandwidth. It is noted that the interference fringes can still be seen for the sample with SF film (S-II) as shown in Fig. 5b. The spontaneous emission peak of both samples, close to the PL peak in Fig. 4a, is located at 521 nm. As pump fluence increases above a certain value, a smaller ASE peak emerges at 546 nm. As pump fluence increases further, the increase rate of the ASE peak intensity is higher than the spontaneous emission. Furthermore, sample S-II reveals a larger increase rate of ASE peak intensity than that without SF. Figure 5c shows the plot of the peak intensity (blue symbol line) and FWHM (red symbol line) as a function of the pump fluence from the emission spectrum of Fig. 5a and b. By the linear fitting of emission peak intensity, the slope efficiency of sample S-II with SF (blue open squares) is higher than that without SF for (S-I, blue solid circles) and there is a great reduction in the lasing threshold from 77.6 μJ/cm$^{2}$ for S-I to 32.7 μJ/cm$^{2}$ for S-II. Here, the threshold of the sample was obtained from the intersection of two lines. The higher slope efficiency and lower threshold for sample S-II is attributed to the regular stack of QDs on the SF film (as shown in the SEM image) and the larger gain cross section.

**Figure 6:**
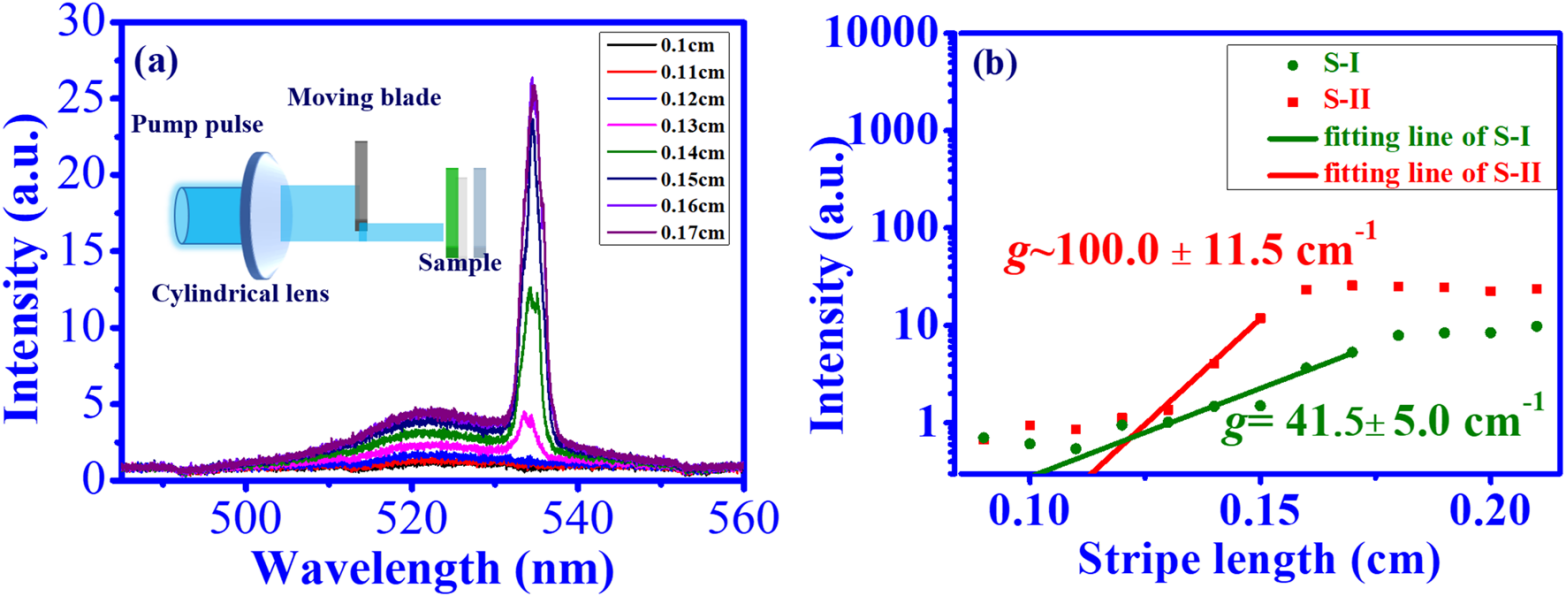
Gain coefficient measurement of CsPbBr_3_ QD films on SF by the VSL model. (**a**) Emission spectrum of CsPbBr_3_ QDs with different stripe lengths (Inset figure: experimental setup of VSL measurement), (**b**) Plot of ASE intensity as a function of stripe length. [Green circles: without SF, red squares: with SF. Solid line: fitting line by Eq. (2)].

In the following, the optical gain of the CsPbBr_3_ QD film was measured by the variable stripe length (VSL) method^40,41^ as shown in the inset of Fig. 6a. Here, the pump beam was focused onto the sample with stripe line ($\sim$ 3.12 mm $\times$ 0.53 mm) using a cylindrical lens with a focal length of about 7.5 cm. Then, we used a moving blade to control the pump stripe length. Lke the previous report^42^, the intensity of the edge-emitted ASE is measured in correspondence to the excitation stripe length. Figure 6a shows the stripe length dependent emission spectra of sample S-II under a pump energy density of 50.6 μJ/cm$^{2}$. The plot of the ASE peak intensity from the two samples (S-I: green circles, S-II: red squares) as a function of the stripe length is shown in Fig. 6b. It is noted that the peak intensity increases sharply when the stripe length is above a certain value, which can be described by^41^

2 $$\begin{aligned} \ I(L)=(I(S)\cdot A/g)[\exp (gL)-1], \end{aligned}$$

where *I*(*L*) is the emission intensity, *g* is the gain efficiency, *I*(*S*) is the spontaneous emission rate per unit volume, *L* is the stripe length and *A* is the cross-sectional area of the excited volume, respectively. By the good fitting of Eq. (2) in Fig. 6b (red and green lines), the optical gain coefficients of CsPbBr$_3$ QDs on the PET substrate without and with SF film are 41.5 cm$^{-1}$ and 100.0 cm$^{-1}$, respectively. With the assistance of SF, the obtained gain coefficient of the CsPbBr_3_ QD/SF film is larger than that on the glass substrate of around 73 cm$^{-1}$^16^. In contrast to sample S-I, the larger gain coefficient for sample S-II is attributed to the regular arrangement and better orientation of aggregated CsPbBr$_3$ QDs on the SF film.

**Figure 7:**
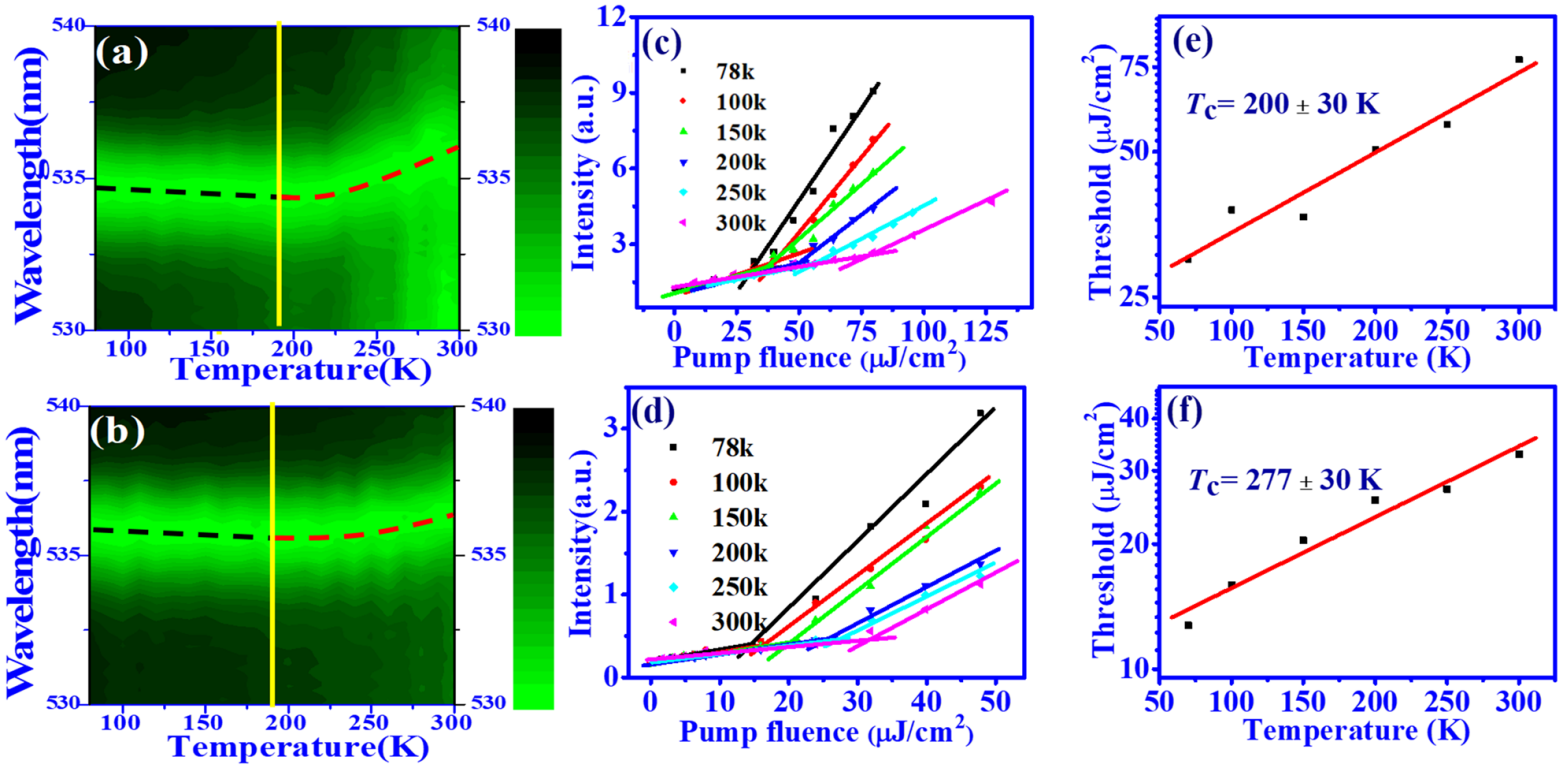
Two-dimensional map of the normalized intensity distribution from temperature-dependent ASE measurement for (**a**) S-I (W/O SF) and (**b**) S-II (W/ SF). ASE peak intensity of (**c**) S-I and (**d**) S-II as a function of pump fluence at different temperatures (78 K to 300 K). Lasing threshold of ASE for samples (**e**) S-I, and (**f**) S-II as a function of temperature and red solid line from fitting of Eq. (3) to obtain the characteristic temperature $T_{c}$.

Figure 7a and b show the two-dimensional (2D) map for the normalized intensity distribution of ASE from samples S-I and S-II as the temperature increases. For S-I, the ASE peak reveals a slight blue-shift of around 0.4 nm (black dashed line) as the temperature increases from 78 to 170 K because of lattice thermal expansion^37^. As the temperature further increases from 170 to 300 K, a slight red-shift of around 1.3 nm (red dashed line) was observed because of exciton-phonon scattering^16^. On the other hand, the shift of the ASE peak with temperature for sample S-II with SF film becomes less apparent, with only a 0.2 nm blue-shift from 78 to 170 K and a 0.9 nm red-shift from 170 to 300 K, respectively. Figure 7c and d show the variation of ASE peak intensity with pump fluence at different operation temperatures. Like previous results^37^, the CsPbBr$_3$ QD film reveals the highest slope efficiency and lowest lasing threshold of around 12.7 μJ/cm$^{2}$ for S-II, or 29.9 μJ/cm$^{2}$ for S-I, in operation at the lowest temperature *T* = 78 K (black line). As the temperature increased to 300 K, the lasing threshold of perovskite increased to 32.7 μJ/cm$^{2}$ for S-II, or 77.6 μJ/cm$^{2}$ for S-I, and the slope efficiency decreased owing to the enhancement of the nonradiative recombination and exciton-phonon scattering^37^.

Figure 7e and f illustrate the variation of the lasing threshold from the CsPbBr$_3$ QD film as a function of temperature, which can be described by^16^

3 $$\begin{aligned} \ P_{th}(T)=P_{th,0}exp({\frac{T}{T_{c}}}), \end{aligned}$$

where $P_{th,0}$ is the threshold of pump fluence at a low temperature, and $T_{c}$ is the characteristic temperature. By the good fitting of Eq. (3), the characteristic temperature of the sample with SF film (S-II) of around 277 ± 30 K is larger than that without SF (S-I) of around 200 ± 30 K, and is even larger than the CsPbBr$_{3}$ QD film on the glass substrate of around 230 30 K^16^. This difference indicates that the ASE from the CsPbBr$_{3}$ QD film with SF is less sensitive to temperature variation. Besides, the characteristic temperature is likely to affected by the trap state above and below the exciton state that depend on the aggregation of QDs^43^. In considering the time dependent emitting population, the activation energy of non-radiation process can also influence the characteristic temperature.

**Figure 8:**
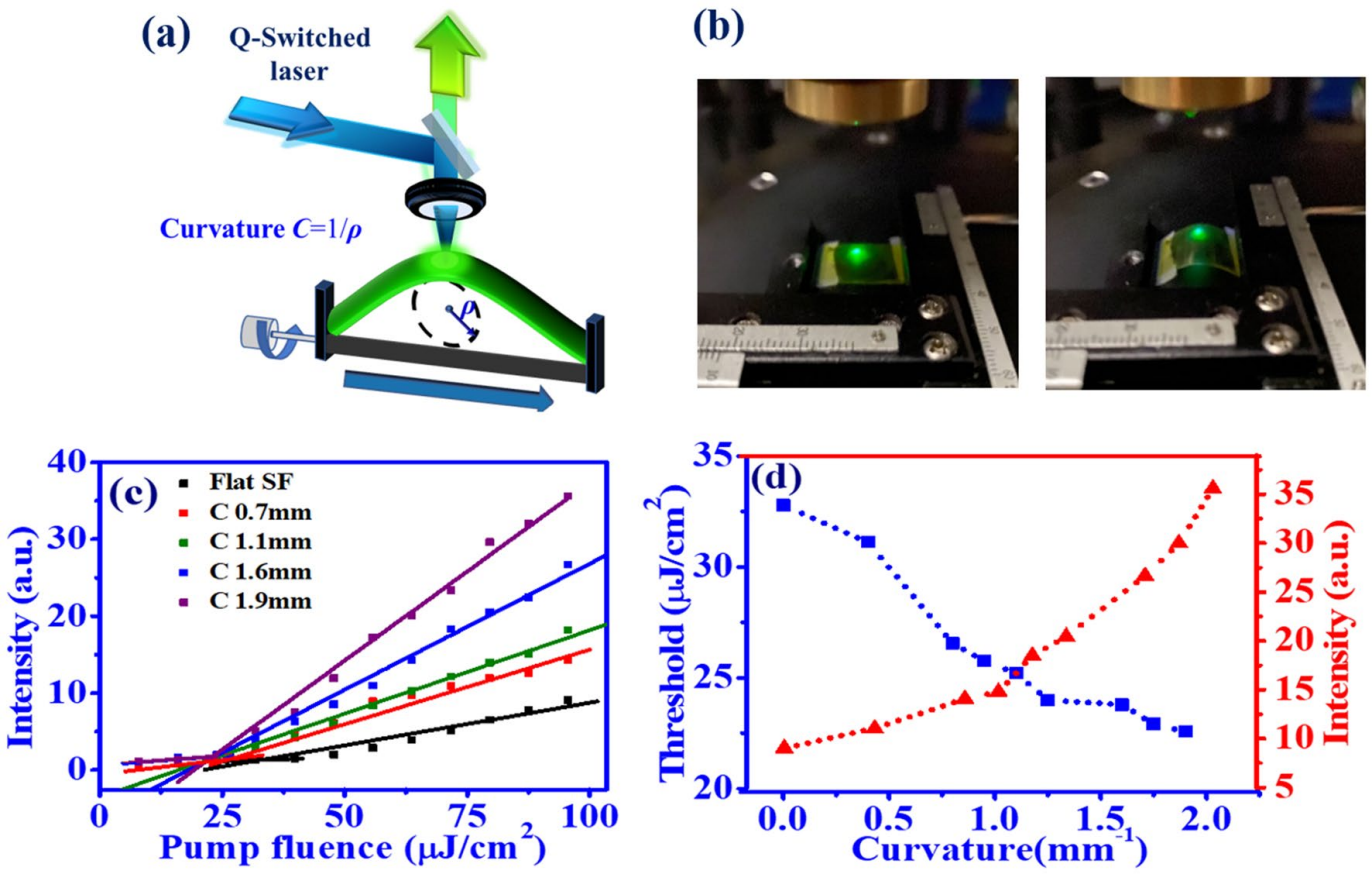
ASE generation of CsPbBr$_3$ QDs excitation by the Q-switched laser under bending condition, (**a**) schematic plot of experimental setup, (**b**) photo of flexible CsPbBr$_3$ QDs/SF film on the PET substrate without bending (Left) and under bending (right) condition. (**c**) Peak intensity as a function of pump fluence with different curvatures of the PET substrate and (**d**) the threshold (blue square) and highest peak intensity (red triangle) of ASE (with pump fluence = 99.54 μJ/cm$^{2}$) as a function of curvature.

Figure 8a shows the experimental setup to investigate the RT ASE behavior from flexible CsPbBr$_3$ QD/SF film under the bending condition (Fig.8b). By the pump of the Q-switched laser, the emission light from the surface normal of the curved sample was collected by a spherical lens and then measured by spectrometer. Figure 8c reveals the ASE peak intensity as a function of pump fluence under different curvatures of perovskite QD films. As the curvature of the bent sample increases, the slope efficiency increases and the lasing threshold decreases. The lasing threshold (blue squares) and the highest output intensity under the pump fluence of 99.54$\mu$J/cm$^{2}$ (red circles) of perovskite film as a function of curvature are illustrated in Fig. 8d. It shows that the threshold declines as the curvature of the sample increases. As the curvature increases to 1.9 mm$^{-1}$, the CsPbBr$_3$ QD film indicates the lowest lasing threshold and the highest ASE peak intensity. The increase in slope efficiency and the decrease in the lasing threshold of the bent sample might be attributed to the enhancement of the index guiding effect. The higher order mode might be filtered out as the curvature of the sample increases. Thus, the mode competition can be reduced to lower the lasing threshold, and can thus increase conversion efficiency.

## Conclusion

In this work, we investigated the amplified spontaneous emission from all-inorganic perovskite QDs on a flexible PET substrate.In contrast to the film deposited directly on top of the PET substrate, the slope efficiency of the CsPbBr$_3$ QDs film increased along with the reduction in the lasing threshold from 77.6 to 32.7 μJ/cm$^{2}$ with the assistance of the silk fibroin (SF) film. By the variable stripe measurement, the CsPbBr$_3$ QDs/SF film reveals a larger gain coefficient of around 100.0 cm$^{-1}$ than that without SF film. The lasing performance improvement of the perovskite film is attributed to the regular alignment of QDs on SF as shown in the SEM image. We also demonstrated that the CsPbBr$_3$ QD/SF film reveals a temperature insensitive property including a smaller lasing peak wavelength shift as the temperature increases and larger characteristic temperature of around 277 K from the precise thermal control ASE measurement. Finally, the reduction in the lasing threshold has also been verified under the bending of the CsPbBr$_3$ QD film because of the index guiding effect. In short, the investigation of the CsPbBr$_3$ QDs film on a flexible substrate with the assistance of the SF film provides a cost-effective and thermal stable strategy to design a bio-compatible laser that possesses relatively high potential for use in bio-imaging in the near future.
